# Bioactive Food Compounds and Human Health: From Dietary Sources to Biological Function

**DOI:** 10.3390/nu18040573

**Published:** 2026-02-09

**Authors:** Enying Cui, Weiwei Cui

**Affiliations:** Department of Nutrition and Food Hygiene, School of Public Health, Jilin University, Changchun 130012, China; cuiey25@mails.jlu.edu.cn

## 1. Introduction

Bioactive food compounds have attracted growing attention for their ability to influence human health beyond basic nutritional functions. A wide range of food-derived constituents, including phytochemicals and bioactive peptides, modulate oxidative stress, inflammation, metabolic regulation, and disease-related biological processes [1,2,3]. These effects arise from complex interactions among dietary components, host physiology, and the gut microbiota [4]. This Special Issue, “Bioactive Food Compounds and Human Health", brings together recent studies that reflect current progress in this field and highlight key challenges in translating mechanistic insights into meaningful health applications.

## 2. Bioactive Food Compounds and Human Health

From a global public health perspective, dietary exposures remain among the most persistent and influential risk factors for disease burden worldwide. Recent Global Burden of Disease analyses indicate that overall dietary risk exposures have shown minimal improvement over the past three decades, with several components remaining at consistently high levels globally. At the same time, metabolic risk factors closely linked to diet, including an elevated body mass index and fasting plasma glucose, have continued to increase [1]. These trends underscore that suboptimal dietary patterns represent a sustained and unresolved challenge rather than a transient risk. Within this context, bioactive food compounds derived from plant-based foods and other functional sources are recognized not merely as nutritional supplements, but as potential modulators of diet-related metabolic and inflammatory pathways, highlighting their relevance to contemporary strategies for chronic disease prevention and health promotion.

Bioactive food compounds act as critical modulators of human health. Their biological effects are highly context-dependent and are shaped by compound structures, food matrix characteristics, processing conditions, and host-specific factors, including metabolic status and microbiome composition [5,6,7].

Specific bioactive food compounds exert well-defined, dose-dependent effects on metabolic, cardiovascular, immune, and gut health. β-Glucans from oats and yeast reduce low-density lipoprotein cholesterol by enhancing bile acid excretion, attenuate postprandial glycemic responses, and activate innate immune signaling through pattern-recognition receptors [8]. Catechins, dominated by epigallocatechin gallate from green tea, activate endogenous antioxidant pathways, inhibit intestinal carbohydrate digestion, and modulate energy metabolism, with reported benefits for adiposity and cardiovascular health [9,10]. Adequate dietary fiber intake improves metabolic homeostasis by delaying nutrient absorption and promoting the microbial production of short-chain fatty acids, thereby supporting intestinal barrier function and systemic metabolic regulation [11,12]. Together, these examples illustrate that bioactive food compounds influence human health through identifiable molecular targets and gut-centered mechanisms that are directly relevant to contemporary strategies for chronic disease prevention.

At the same time, advances in food science have expanded the functional landscape of bioactive compounds. Strategies such as molecular complexation, encapsulation, and matrix optimization have been developed to improve stability, bioaccessibility, and biological efficacy without fundamentally altering dietary patterns [13,14]. In parallel, a growing interest in non-traditional and sustainable food sources has drawn attention to bioactive peptides and other functional components derived from edible insects and alternative protein systems, further broadening the scope of bioactive food research [15,16,17].

These developments reflect a broader shift in nutrition science from reductionist descriptions of individual compounds toward mechanism-driven and systems-oriented approaches. Clarifying how bioactive food compounds interact with the gut environment, microbial metabolism, and host signaling networks is essential for translating experimental findings into robust dietary strategies and evidence-based health applications.

## 3. Overview of the Contributions in This Special Issue

Chen et al. (2025) investigated a soybean lecithin–gallic acid complex as a means of enhancing radiosensitivity in non-small-cell lung cancer cells. The study demonstrates that improving the bioavailability of gallic acid through complex formation promotes ferroptosis via redox-related signaling pathways, including the Nrf2/SLC7A11/GPX4 axis. This work illustrates how formulation strategies can amplify the biological efficacy of food-derived polyphenols and supports the potential integration of bioactive food compounds into adjunctive cancer therapy approaches (Contribution 1).

Praseatsook et al. (2025) explored bioactive peptides derived from black soldier fly larvae and evaluated their antioxidant, anti-inflammatory, antimutagenic, and anticancer activities. They highlight edible insects as sustainable and underexplored sources of functional peptides, expanding the scope of bioactive food compounds beyond traditional plant- and animal-derived ingredients (Contribution 2).

Hayden et al. (2025) investigated the effects of cocoa polyphenols on gut microbial communities at different intestinal sites using a mouse model of colitis. The results demonstrate that cocoa polyphenols modulate the gut microbiome in a site-specific manner, with distinct responses observed across luminal and mucosa-associated compartments. Their findings emphasize the spatial heterogeneity of the intestinal ecosystem and underline the importance of the sampling location when assessing the impact of dietary bioactive compounds on gut health (Contribution 3).

Zhang et al. (2026) applied integrated 16S rRNA sequencing and metabolomics to evaluate the protective effects of (E)-flavokawain A in an AOM/DSS-induced colorectal cancer model. By linking microbial alterations with metabolic changes, this work strengthens the conceptual link between dietary phytochemicals, microbial metabolites, and colorectal cancer prevention (Contribution 4).

Kurhaluk et al. (2025) focused on phenolic compounds and terpenes in plant-based foods, highlighting their combined influence on sensory characteristics and health-related biological functions. By integrating evidence from food chemistry, sensory science, and nutrition research, this work emphasizes that phytochemicals simultaneously shape flavor perception and contribute antioxidant, anti-inflammatory, and anticancer activities. The review also discusses the impact of food processing and matrix effects on compound stability and bioavailability, underscoring the importance of considering technological and sensory factors when evaluating the functional value of bioactive food compounds (Contribution 5).

Wang and Cui (2026) examined the role of phytochemicals in atherosclerosis through the gut–liver axis. This contribution synthesizes current evidence demonstrating that bioactive food compounds modulate lipid metabolism, bile acid signaling, intestinal barrier integrity, and inflammatory responses via interactions with the gut microbiota. By framing cardiovascular disease within a diet–microbiota–host interaction model, the review advances a systems-level understanding of how dietary bioactives influence atherosclerotic processes and highlights microbial metabolism as a key determinant of phytochemical bioactivity (Contribution 6).

## 4. Future Perspectives

Advancing the field of bioactive food compounds and human health will require integrated, systems-oriented research strategies. Future studies should combine multi-omics technologies, spatial analyses, and longitudinal designs to capture the dynamic interactions between diet, the gut microbiota, and host physiology. Improving delivery strategies and food formulations may also help overcome bioavailability barriers, enhancing the functional efficacy of bioactive compounds in realistic dietary settings.

Equally important is the translation of mechanistic insights into clinical and public health contexts. Carefully designed human studies are needed to define effective intake ranges, identify responsive populations, and evaluate long-term safety. A clear communication of scientific evidence, supported by regulatory consistency, will be essential to bridge the gap between research advances and consumer trust.

## 5. Conclusions

The contributions to this Special Issue collectively highlight the diverse and interconnected mechanisms through which bioactive food compounds influence human health. Rather than acting through single molecular targets, these compounds modulate complex networks involving food matrices, gut microbiota, metabolic pathways, and cellular stress responses. By bridging food science, microbiome research, and disease biology, *Bioactive Food Compounds and Human Health* provides a timely perspective on current progress and future challenges in the field. Continued interdisciplinary efforts will be critical for translating mechanistic insights into credible and effective nutritional strategies for chronic disease prevention and health promotion.
